# The Efficacy of Palliative Care Education and Training in Primary Care Settings: A Scoping Review of the Literature

**DOI:** 10.1093/geroni/igaa057.1443

**Published:** 2020-12-16

**Authors:** Jessica Hsieh, Raza Mirza, Lynn McDonald, Christopher Klinger

**Affiliations:** 1 University of Toronto, Toronto, Ontario, Canada; 2 National Initiative for the Care of the Elderly, Toronto, Ontario, Canada

## Abstract

Primary care providers play an important role in palliative care service provision. A scoping review of the literature was undertaken in an attempt to gain insight into and awareness of palliative education and training needs in primary care settings. Four scientific databases covering the medical and social science fields were searched, alongside Grey literature. A total of 5,109 hits were generated, leading to 2,875 titles for individual review. Of these, 33 articles were included in the final review. Five major themes were identified: (1) communication skills; (2) knowledge of spiritual/psychosocial needs; (3) pain and symptom management; (4) cultural proficiency; and (5) experience working within interdisciplinary teams. Many primary care practitioners felt inadequately trained in palliative care and felt unprepared to provide the necessary care. Specifically, poor communication between healthcare professionals and patients were found to adversely affect the level of palliative care that is provided. Additionally, practical experience in palliative/end-of-life care was cited as one of the most beneficial methods in helping to improve knowledge about and ability to practice in the field. The majority of articles emphasized the need for education and training programs to enhance the quality of palliative/end-of-life care service provision. Palliative care education appeared to have numerous benefits, including enhancing providers’ knowledge of and attitudes towards this subject, ability to provide palliative/end-of-life care, and self-perception of preparedness. As the landscape of education needs are constantly changing, this review serves as one of the steps in an ongoing evaluation of palliative care providers’ training needs.

